# P-332. Screening Practices for HIV and Sexually Transmitted Infections During Cabotegravir Long Acting or Daily Oral Pre-Exposure Prophylaxis Use in the US

**DOI:** 10.1093/ofid/ofaf695.551

**Published:** 2026-01-11

**Authors:** Steven K Barnett, Laurence Brunet, Jennifer S Fusco, Karam Mounzer, Kevin R Frost, Supriya Sarkar, Kimberley Brown, Harmony Garges, Vani Vannappagari, Gregory P Fusco

**Affiliations:** CAN Community Health, Tampa, FL; Epividian, Inc., Durham, North Carolina; Epividian, Inc., Durham, North Carolina; Philadelphia Fight Community Health Centers, Philadelphia, Pennsylvania; amfAR, The Foundation for AIDS Research, New York, New York; ViiV Healthcare, Baltimore, Maryland; ViiV Healthcare, Baltimore, Maryland; ViiV Healthcare, Baltimore, Maryland; ViiV Healthcare, Baltimore, Maryland; Epividian, Inc., Durham, North Carolina

## Abstract

**Background:**

HIV testing is required before pre-exposure prophylaxis (PrEP) initiation and recommended before an oral PrEP prescription refill or at the time of a cabotegravir long acting (CAB LA) PrEP injection per the CDC PrEP guidelines. Regular screening for syphilis, gonorrhea, and chlamydia are also recommended, with frequency based on risk characteristics. We describe HIV and sexually transmitted infection (STI) testing practices during CAB LA or oral PrEP use in the OPERA cohort.

**Figure:**
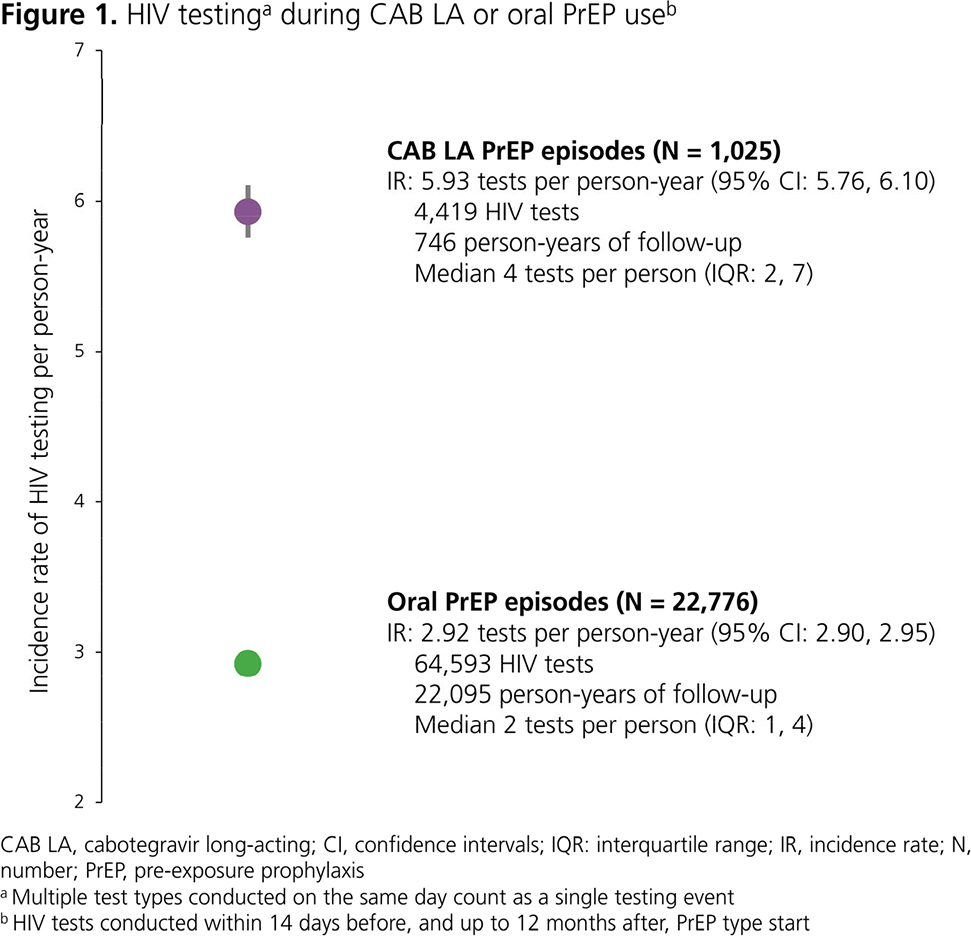

**Figure:**
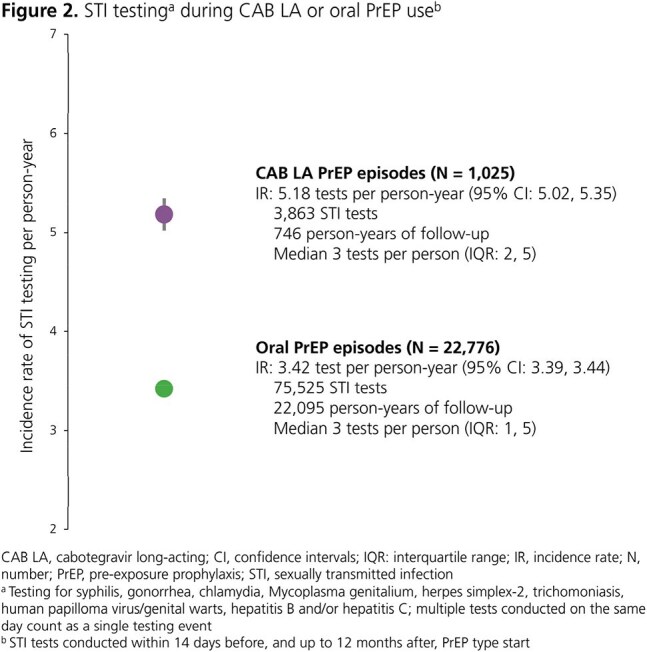

**Methods:**

Adults without HIV who received ≥ 1 CAB LA injection and/or an oral PrEP prescription in the OPERA cohort (21DEC2021-30JUN2023) were followed through 30JUN2024. Individuals could contribute to multiple PrEP episodes, defined as continuous use of a specific PrEP type (CAB LA, oral). Incidence rates (IR) of HIV and STI testing (univariate Poisson regression) and proportions of individuals receiving HIV or STI testing at relevant intervals were estimated from 14 days before, up to 12 months after, PrEP episode start.

**Figure:**
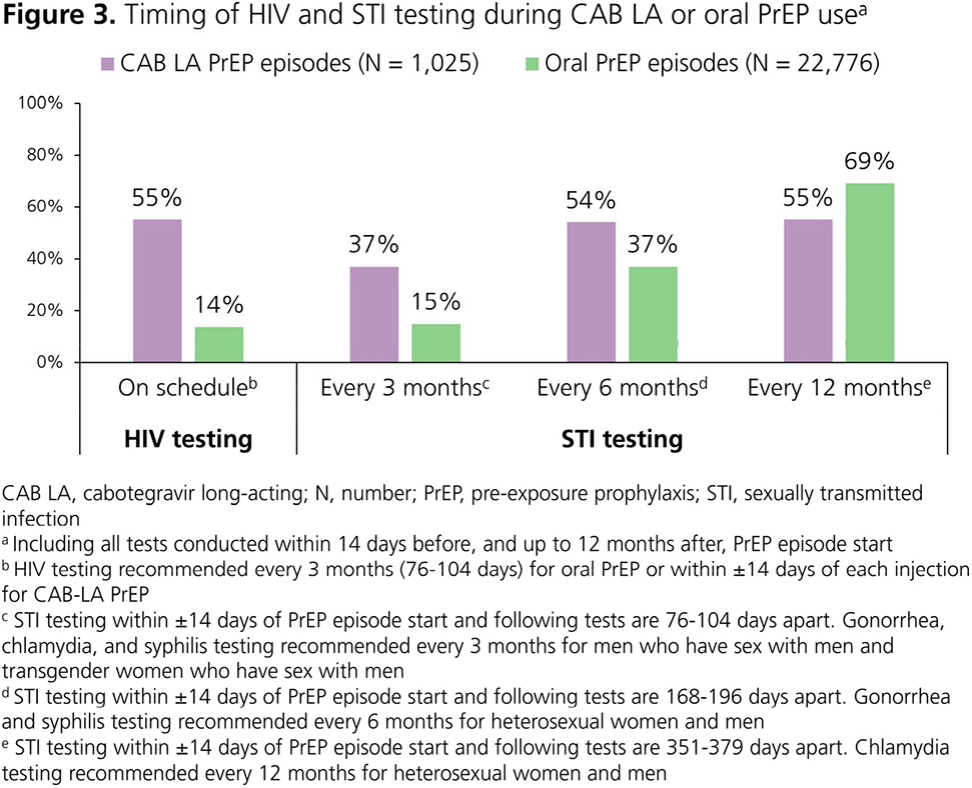

**Results:**

A total of 1,025 CAB LA PrEP episodes and 22,776 oral PrEP episodes were included. HIV testing occurred twice as frequently on CAB LA PrEP (IR: 5.93; 95% confidence interval [CI]: 5.76, 6.10) than oral PrEP (IR: 2.92; 95% CI: 2.90, 2.95) (Fig 1). Similarly, STI testing occurred 1.5 times more frequently on CAB LA PrEP (IR: 5.18; 95% CI: 5.02, 5.35) than oral PrEP (IR: 3.42; 95% CI: 3.39, 3.44) (Fig 2). Both HIV and STI testing were more likely to occur at the predefined intervals during CAB LA PrEP than oral PrEP episodes (Fig 3). The difference was most pronounced for HIV testing (55% vs 14%) and STI testing every 3 months (37% vs 15%). The 12-month HIV incidence rates per 100 person-years were 0.18 (95% CI: 0.03, 1.26) for CAB LA and 0.59 (95% CI: 0.50, 0.71) for oral PrEP.

**Conclusion:**

HIV PrEP clinical care represents an opportunity to provide sexual health-related services. In this large, real-world US cohort of PrEP users, HIV acquisition rates were low. However, HIV testing was suboptimal, raising concerns for delayed HIV diagnosis and treatment. Rates of both HIV and STI testing were significantly higher during CAB LA PrEP use than oral PrEP use. These results suggest that increased clinical contact from regular CAB LA PrEP injection visits may encourage more frequent HIV and STI testing among PrEP users.

**Disclosures:**

Laurence Brunet, PhD, Gilead Sciences: Grant/Research Support|Merck & Co.: Grant/Research Support|Theratechnologies Inc.: Grant/Research Support|ViiV Healthcare: Grant/Research Support Jennifer S. Fusco, BS, Gilead Sciences: Grant/Research Support|Merck & Co.: Grant/Research Support|Theratechnologies Inc: Grant/Research Support|ViiV Healthcare: Grant/Research Support Karam Mounzer, MD, Aspire Scientific: Medical writing support|Gilead Sciences: Advisor/Consultant|Gilead Sciences: Grant/Research Support|Gilead Sciences: Honoraria|Gilead Sciences: Medical writing support Supriya Sarkar, PhD, MPH, ViiV Healthcare: Stocks/Bonds (Public Company) Kimberley Brown, PharmD, ViiV Healthcare: Employee|ViiV Healthcare: Stocks/Bonds (Public Company) Harmony Garges, MD, GSK: I am an employee of ViiV Healthcare|GSK: Stocks/Bonds (Public Company) Vani Vannappagari, MBBS, MPH, PhD, ViiV Healthcare: Full time Employee of ViiV Healthcare and owns GSK stock|ViiV Healthcare: Stocks/Bonds (Public Company) Gregory P Fusco, MD, MPH, Gilead: Grant/Research Support|Merck: Grant/Research Support|Theratechnologies: Grant/Research Support|Viiv: Grant/Research Support

